# AI-Based Augmented Reality Microscope for Real-Time Sperm Detection and Tracking in Micro-TESE

**DOI:** 10.3390/bioengineering13010102

**Published:** 2026-01-15

**Authors:** Mahmoud Mohamed, Ezaki Yuriko, Yuta Kawagoe, Kazuhiro Kawamura, Masashi Ikeuchi

**Affiliations:** 1 Laboratory for Biomaterials and Bioengineering, Institute of Science Tokyo, Tokyo 152-8550, Japan; mahmoud.mohamed.abda@tmd.ac.jp (M.M.); kawagoe.y.91128@gmail.com (Y.K.); 2 Graduate School of Information Science and Technology, The University of Tokyo, Tokyo 113-8654, Japan; 3 Departments of Obstetrics and Gynecology, Juntendo University Faculty of Medicine, Tokyo 113-8421, Japan; k.kawamura.aq@juntendo.ac.jp; 4 Departments of Obstetrics and Gynecology, International University Health and Welfare School of Medicine, Chiba 286-8686, Japan

**Keywords:** augmented reality microscope, non-obstructive azoospermia, Micro-TESE, sperm detection, deep learning, object tracking, sperm motility analysis

## Abstract

Non-obstructive azoospermia (NOA) is a severe male infertility condition characterized by extremely low or absent sperm production. In microdissection testicular sperm extraction (Micro-TESE) procedures for NOA, embryologists must manually search through testicular tissue under a microscope for rare sperm, a process that can take 1.8–7.5 h and impose significant fatigue and burden. This paper presents an augmented reality (AR) microscope system with AI-based image analysis to accelerate sperm retrieval in Micro-TESE. The proposed system integrates a deep learning model (YOLOv5) for real-time sperm detection in microscope images, a multi-object tracker (DeepSORT) for continuous sperm tracking, and a velocity calculation module for sperm motility analysis. Detected sperm positions and motility metrics are overlaid in the microscope’s eyepiece view via a microdisplay, providing immediate visual guidance to the embryologist. In experiments on seminiferous tubule sample images, the YOLOv5 model achieved a precision of 0.81 and recall of 0.52, outperforming previous classical methods in accuracy and speed. The AR interface allowed an operator to find sperm faster, roughly doubling the sperm detection rate (66.9% vs. 30.8%). These results demonstrate that the AR microscope system can significantly aid embryologists by highlighting sperm in real time and potentially shorten Micro-TESE procedure times. This application of AR and AI in sperm retrieval shows promise for improving outcomes in assisted reproductive technology.

## 1. Introduction

In Japan, the trends of later marriage and lower birth rates have continued to accelerate alongside the diversification of lifestyles. The average age at first marriage is now 31.0 years for men and 29.4 years for women, which represents an increase of 2.5 years for men and 3.1 years for women compared to 25 years ago [1]. The age at which women have their first child has also risen: in 2019, the average age of a mother at the birth of her first child was 30.7 years, about 4 years older than it was 44 years prior. At the same time, it is well known that fertility (the ability to conceive or cause conception) decreases with advancing age in both men and women.

Assisted Reproductive Technology (ART) is defined as “all treatments or methods that involve handling human oocytes (eggs) and sperm, or embryos, with the aim of achieving a pregnancy” [2]. Generally, ART is an umbrella term for infertility treatments including in vitro fertilization and embryo transfer (IVF-ET), intracytoplasmic sperm injection and embryo transfer (ICSI-ET), cryopreservation and thawed embryo transfer, and related procedures. In 2022, Japan saw a significant rise in ART usage, with over 543,000 registered cycles and more than 77,000 live births—a 9–10% increase from the previous year. Notably, 57.5% of oocyte retrieval cycles were freeze-all, and frozen–thawed embryo transfers (FET) led to over 72,000 births. The average patient age was 37.6 years, with nearly 39% of cycles involving women aged 40 or older. High single embryo transfer rates (over 82%) contributed to singleton live birth rates exceeding 96%, indicating effective and safer ART practices [3]. Therefore, demand driven by technological advances in assisted reproductive technology is significant and is projected to continue expanding.

One particular infertility treatment addressing male factor infertility is Microdissection Testicular Sperm Extraction (Micro-TESE). Micro-TESE is a surgical procedure, primarily used for patients with non-obstructive azoospermia (NOA), in which a surgeon retrieves sperm directly from testicular tissue under an operating microscope [4]. Micro-TESE is a highly specialized procedure that often requires a long time, ranging from about 1.8 up to 7.5 h to complete. One reason the procedure can be so time-consuming is the uncertainty involved in identifying sperm within the excised tissue. The surgeon and embryologist must determine whether no sperm are present in a given sample or whether sperm were present but simply missed (overlooked) by the embryologist’s search. This process may require examining multiple tissue samples until sperm are found, which can be laborious. Furthermore, Micro-TESE is physically and mentally taxing for all involved. The patient must remain under anesthesia while the surgeon and embryologist painstakingly search for potentially scarce sperm cells. If the embryologist fails to find sperm in the initial samples, the surgeon may have to retrieve additional tissue samples, prolonging the surgery. Each additional sample and extended search increases tissue damage and the duration of anesthesia, as well as the overall stress on the patient and medical team.

Augmented Reality (AR) refers to a technology that superimposes digital content such as navigational markers, 3D data, and video onto real-world scenes. This can be achieved using dedicated head-mounted displays or by utilizing the camera and display of a smartphone. AR technologies have become increasingly widespread across industries, including entertainment, technical training, and industrial applications.

In the medical field, the adoption of AR is also expanding. An integrated system known as the Augmented Reality Microscope (ARM) has been proposed, which combines machine-learning models for cancer diagnosis with an AR-enabled optical microscope [5]. ARM captures the microscope’s field of view through the eyepiece in real time and overlays diagnostic information directly onto the user’s visual field. As the specimen images are continuously acquired, deep-learning algorithms process each frame and generate inference outputs (e.g., heatmaps). These outputs are then post-processed to ensure that the overlaid information is informative while not obscuring the underlying specimen. Examples include colored contour overlays to support detection and diagnosis, as well as text labels indicating measurements such as size. ARM demonstrates its usefulness with two cancer-detection algorithms: one for identifying metastatic breast cancer in lymph-node samples, and another for detecting prostate cancer in prostatectomy specimens. The implementation operates at approximately 10 frames per second (FPS), enabling seamless updates even when the user moves the slide or changes magnification. Because the platform can be retrofitted to existing optical microscopes using low-cost components and does not require full slide digitization, it has the potential to accelerate the global deployment of deep-learning tools among pathologists. Optical microscopes are widely used not only in pathology but also in numerous scientific and industrial fields; thus, ARM is expected to find applications across healthcare, life-science research, and materials science.

Detecting sperm in Micro-TESE specimens poses distinct challenges not found in ejaculate samples [6,7]. These include the presence of pre-spermatogenic cells in seminiferous tubules, lack of sperm motility (which prevents motion-based detection), and the clinical restriction on staining methods.

Earlier work utilized AKAZE feature extraction [8] and MB-LBP descriptors [9], classifying patches with AdaBoost [10]. This achieved high recall (0.87) but low precision (0.39) and high computational cost due to dense keypoint extraction [11].

To address this, our previous work proposed SD-CLIP (Sperm Detection using Classical Image Processing) [12], a lightweight, morphology-based method using Sobel filtering, connected-component labeling, and PCA. It identified elliptical heads and thin tails, reducing detection candidates (48 vs. 1970) and processing time (72 ms vs. 275 ms) compared to AKAZE-based methods. However, its performance remained sensitive to imaging conditions and required manual tuning.

**Figure 1:**
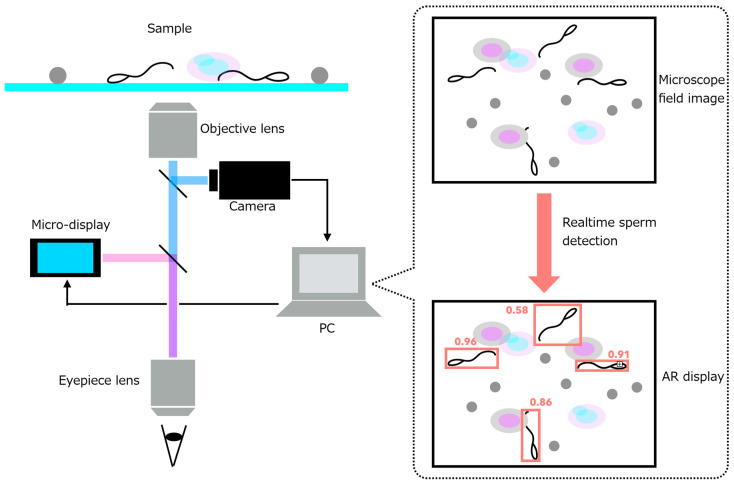
Concept of the proposed system.

In this study, we extend beyond algorithmic improvements by introducing a deep-learning-based sperm detection framework integrated into a real-time AR microscope system. We develop a system that accelerates the microscopic search and retrieval of fertilization-competent sperm during Micro-TESE procedures. To address the reliance on operator expertise and the prolonged search time associated with conventional manual microscopy, we propose an integrated assistance system that combines an AR microscope with real-time sperm-analysis software. The system captures microscopic images of testicular tissue, automatically detects spermatozoa, and overlays their positional information directly within the microscope’s field of view using a micro-display-based AR interface (Figure 1). To realize this concept, we develop two core components:

(1) an AR microscope capable of extracting image features from a high-speed camera feed and projecting them into the visual path at interactive rates, and
(2) a real-time sperm-analysis module that performs sperm detection, tracking, and motility-related speed analysis.

The design of the proposed system is guided by key performance requirements: avoiding mechanical or phototoxic damage to sperm, reducing the time needed for microscopic search, achieving high precision and recall despite the low prevalence of sperm in testicular tissue, improving overall sperm detection rates, and enabling the selection of morphologically and kinematically favorable sperm comparable to the judgment of an expert embryologist. In parallel, the system must adhere to applicable domestic and international medical regulations, while keeping manufacturing and operational costs within practical limits for eventual clinical deployment.

By meeting these functional and regulatory criteria, the proposed AR-assisted Micro-TESE workflow has the potential to shorten operative time, lessen the burden on embryologists and clinicians, and improve sperm-retrieval opportunities for patients undergoing infertility treatment. Unlike handcrafted methods, our approach enables robust, full-field sperm detection and tracking under variable clinical conditions.

## 2. Materials and Methods

### 2.1. Sample Preparation

The provisional Micro-TESE specimens were prepared as follows.

**Figure 2:**
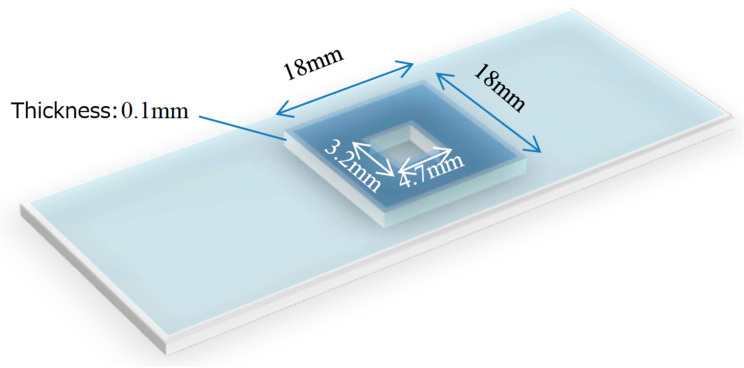
Schematic of the prepared microscope slide for the sperm detection test (A commercially available glass slide is fitted with a silicone rubber frame that fixes the depth of the liquid sample. The sample, containing testicular tissue and a possibly small number of sperm, is placed within the frame. A cover slip is placed on top to seal the sample. This setup ensures a consistent volume and area to examine).

(1) Prepare a cell suspension: thaw frozen HepG2 (human liver cancer-derived) cells and add 1 mL of PBS (−).
(2) Transfer 1 mL of the cell suspension into an Eppendorf tube, add 10 µL of human sperm suspension (derived from healthy donor semen), and mix thoroughly.
(3) Deposit 5 µL of the mixture onto a partitioned slide glass (Figure 2).

### 2.2. Requirements and System Design of the AR Microscope for Sperm Retrieval Support

To develop an AR-assisted microscope system for supporting sperm retrieval, we first defined the technical requirements that the system must satisfy.

The key specifications are as follows:

(1) Response time from acquisition of the microscope image to AR overlay projection must be within 200 ms.
(2) When using a 10× objective lens, the imaging system must resolve the size of spermatozoa (total length: ~50 µm; head short axis: ~3 µm; tail diameter: 0.2–0.3 µm).
(3) The sperm-analysis software must operate on a portable notebook PC, enabling deployment within clinical environments such as operating rooms.

Requirement (1) ensures real-time performance; excessive latency leads to misalignment between the microscope’s optical image and the AR projection. Requirement (2) ensures that the camera input has sufficient resolution for the reliable identification of sperm. Requirement (3) reflects the need for portability in clinical settings and aligns with the cost constraints, maintaining reasonable manufacturing costs. Accordingly, GPU resources integrated into a standard notebook PC are used for sperm-analysis computation.

To realize these requirements, the proposed AR microscope integrates two custom modules into a conventional optical microscope:

• A high-resolution camera module that captures the current microscopic field of view.
• A micro-display module that overlays digital information directly onto the original optical path.

**Figure 3:**
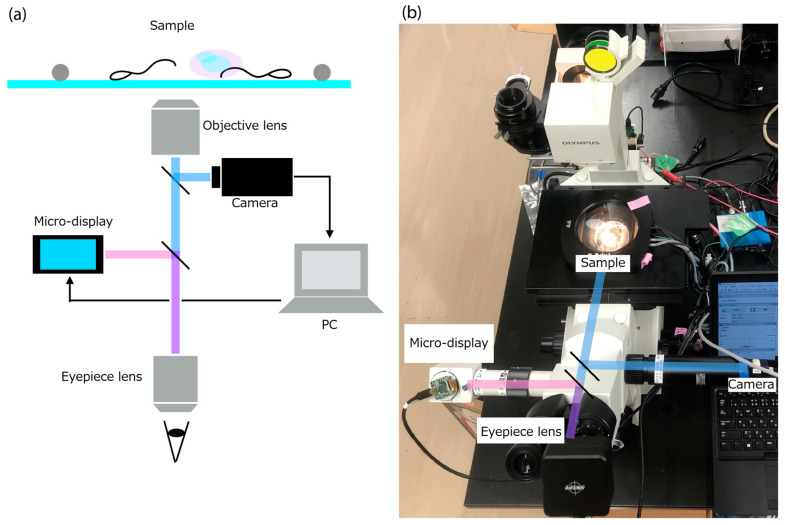
Optical system of the AR microscope system. (**a**) System overview. (**b**) System appearance.

Because only two modules are added to an existing microscope, the system meets the manufacturing and operational cost requirements. The optical configuration is shown in Figure 3. To ensure that the displayed sperm-location predictions coincide precisely with the user’s visual field, the micro-display overlays information on the same plane as the captured image (Figure 3a). The positional correspondence between the camera and display coordinates is established in software after fixing the physical positions and angles of the camera and display modules.

The hardware components used to construct the AR microscope system are listed below:

• Inverted fluorescence microscope: IX70 (Olympus, Hachioji, Japan)
• High-speed USB camera: STC-MBS510U3V (OMRON SENTECH, Ebina, Japan)
  ○ Frame rate: 75.7 FPS
  ○ Effective pixels: 2448 × 2048 (grayscale)
• Micro-display: ECX334C (Sony, Minato-ku, Japan)
  ○ Refresh rate: 57.942 Hz
  ○ Resolution: 1024 × 768 (RGB)
  ○ Maximum brightness: 1000 cd/m^2^
  ○ Contrast ratio: 100,000:1

All components possess sufficient performance for real-time operation and can easily satisfy the target response time of 100–200 ms. Regarding Requirement (2), the imaging scale at 10× magnification is approximately 0.14 µm/pixel, and even with 2× horizontal binning it becomes 0.27 µm/pixel, which remains adequate for resolving the sperm tail diameter (0.2–0.3 µm).

### 2.3. Sperm Detection Model

When detecting motile sperm in semen samples, frame-to-frame image differencing can be used to estimate sperm positions relatively easily. However, sperm present in testicular tissue during Micro-TESE exhibit extremely low motility and often remain nearly stationary across consecutive frames. As a result, motion-based detection methods are not applicable. Therefore, in this study, sperm detection is performed from single-frame images. In addition, because the proposed system must operate in real time for intraoperative AR visualization, the sperm detection model must achieve both high speed and high accuracy. Several approaches can be considered for presenting sperm locations in microscopic images. In general computer vision, the major image-recognition tasks can be ranked by complexity as follows:

(1) Image classification: Identifying only the object category in an image
(2) Object detection: Estimating the positions and classes of multiple objects using bounding boxes
(3) Semantic segmentation: Assigning a class label to each pixel

In ARM-based cancer detection systems [5], semantic segmentation is used to visualize the contours of cancerous cells within microscope images. However, the purpose of the present study is to indicate sperm locations to embryologists, not to visualize precise sperm contours. Therefore, we adopt an object detection framework that outputs bounding boxes indicating sperm positions. Furthermore, to support sperm quality assessment, the system measures sperm displacement and displays motility-related information in the microscope field of view, enabling prioritization during sperm retrieval.

Object detection estimates the location and class of target objects in an image using bounding boxes. It is widely applied in autonomous driving, robotics, security, and medical imaging. Before the advent of deep learning, object detection relied on handcrafted features such as SIFT [13], SURF [14], HOG, Haar-like features [15], and MB-LBP [9], combined with sliding-window search strategies. However, exhaustive scanning over all possible positions and scales resulted in extremely high computational cost.

With the emergence of deep learning, region-based CNNs (R-CNN) [16] were proposed to first extract candidate object regions and then classify them using CNNs. Although R-CNN achieved high detection accuracy on the PASCAL VOC dataset [17], its multi-stage training process and slow inference speed motivated the development of faster variants such as Fast R-CNN [18] and Faster R-CNN [19].

For real-time applications, end-to-end object detection models such as YOLO (You Only Look Once) [20] and SSD (Single Shot MultiBox Detector) [21] were subsequently introduced. These models perform detection and classification in a single forward pass, enabling high-speed inference.

Because sperm size variation is minimal and real-time performance is essential in Micro-TESE support, the present study adopts YOLOv5 [22] to construct the sperm detection model. By using YOLO-based detection instead of staining-based techniques, the proposed method satisfies the clinical requirement of avoiding physical or chemical damage to sperm.

The overall deep learning pipeline used in this study follows a sequential flow. Microscopic images are first acquired through the augmented-reality microscope system and processed in real time by the YOLOv5-based detector to identify sperm candidates in each frame. Detected sperm are then associated across consecutive frames using an online multi-object tracking algorithm, enabling stable tracking and trajectory estimation. Finally, the detection and tracking results are overlaid onto the optical microscope view via the AR interface, providing real-time visual guidance to the operator during Micro-TESE procedures.

### 2.4. Evaluation of Sperm Motility

To improve the quality of sperm used for fertilization, several manufacturers have developed Computer-Aided Sperm Analysis (CASA) systems [23]. These systems can measure sperm motility in semen and, in some cases, also estimate sperm concentration and perform semi-automatic morphological assessment. CASA systems offer two major advantages over manual visual evaluation:

(1) higher measurement precision, and
(2) quantitative acquisition of kinematic parameters such as progressive motility, hyperactivation, and capacitation-related changes.

**Figure 4:**
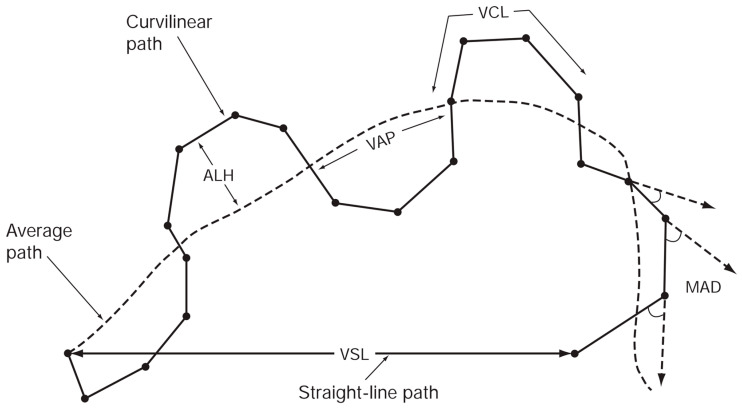
Standard terminology for variables measured by the CASA system [30]. Reproduced with permission from World Health Organization, 2010. Licensed under Attribution-NonCommercial-ShareAlike 3.0 IGO (CC BY-NC-SA 3.0 IGO).

Several studies have reported significant associations between CASA-derived parameters of progressively motile sperm and fertilization rates in vitro and in vivo, as well as the time required to achieve fertilization [24,25,26]. On the other hand, it is known that CASA systems can have difficulty distinguishing sperm heads from debris in samples with low sperm concentration [27,28]. Therefore, it is considered challenging to apply CASA systems, originally designed for semen evaluation, directly to sperm in testicular tissue obtained during Micro-TESE. CASA systems are best suited for kinetic analysis of motile cells, and are thus widely used for sperm kinematic assessment. Standard terminology for motility parameters measured by CASA is summarized in Figure 4 and includes:

(1) VCL (curvilinear velocity, µm/s): Time-averaged velocity of the sperm head along the actual curved trajectory observed in two dimensions under the microscope; an indicator of cellular vigor.
(2) VSL (straight-line velocity, µm/s): Time-averaged velocity along the straight line connecting the first and last detected head positions.
(3) VAP (average path velocity, µm/s): Time-averaged velocity along a smoothed average trajectory connecting the initial and final positions. The average path is obtained by smoothing the curved trajectory according to proprietary CASA algorithms, which can differ among systems and therefore may limit inter-system comparability.
(4) ALH (amplitude of lateral head displacement, µm): Magnitude of lateral deviation of the sperm head from its average path, expressed as the maximum or mean displacement. Because algorithms differ among CASA systems, ALH values may not be directly comparable between systems.
(5) LIN (linearity): Straightness of the curvilinear path, defined as VSL/VCL.
(6) WOB (wobble): Degree of oscillation of the actual trajectory around the average path, defined as VAP/VCL.
(7) STR (straightness): Straightness of the average path, defined as VSL/VAP.
(8) BCF (beat-cross frequency, Hz): Average frequency at which the curvilinear path crosses the average path.
(9) MAD (mean angular displacement, degrees): Time-averaged absolute instantaneous turning angle of the sperm head along its curved trajectory.

### 2.5. Sperm Tracking Model

Multi-Object Tracking (MOT) is an image-processing technique that continuously tracks multiple moving objects in a video sequence while maintaining their identities over time. MOT is an application of object detection to video and is more challenging than static image recognition because objects undergo continuous changes in position, rotation, and appearance. Traditionally, MOT has been implemented by combining methods such as Kalman filters, particle filters, SORT (Simple Online and Realtime Tracking) [29], and optical flow. However, these approaches are known to struggle in situations where objects are temporarily occluded or when objects intersect. Because only the predicted bounding-box positions are considered, object IDs may be lost if an object reappears outside the Kalman filter’s predicted region after occlusion, or ID switches may occur when objects cross paths.

To address these limitations, DeepSORT [31], a tracking model that incorporates appearance similarity, was proposed. DeepSORT is based on the idea that tracking accuracy can be improved by considering visual feature similarity in addition to positional information. In DeepSORT, a convolutional neural network is pre-trained on a large-scale person re-identification dataset (person-ReID) [32], which contains over 1.1 million images of 1261 pedestrians. This network learns discriminative appearance features. For each bounding box detected by the object detection model, a feature vector is extracted using the trained CNN. The cosine similarity between this feature and the historical features of previously tracked objects is then used when associating detections with existing track IDs.

Associations between detections and tracks are computed using the Hungarian algorithm, and the appearance features of matched detections are stored in the track history. DeepSORT uses a Matching Cascade strategy that preferentially associates detections with tracks that have been observed most recently. DeepSORT offers two main advantages:

• high-speed operation, and
• robust tracking even when objects temporarily disappear behind occlusions and then reappear.

Although DeepSORT is somewhat slower than SORT, the incorporation of appearance features allows IDs to be correctly re-associated after short periods without observation.

In the present study, we propose to track spermatozoa within the microscope field of view using DeepSORT. This enables continuous tracking of each sperm and estimation of motion-related parameters. Based on these tracking results, the proposed system can provide a recommendation function that highlights the most motile spermatozoa.

### 2.6. Sperm Velocity Analysis

Using DeepSORT, each sperm is assigned a tracking ID, enabling continuous tracking over time. To implement a recommendation function for selecting high-quality sperm, it is necessary not only to track sperm but also to evaluate and quantify the motility of each tracked spermatozoon.

**Figure 5:**
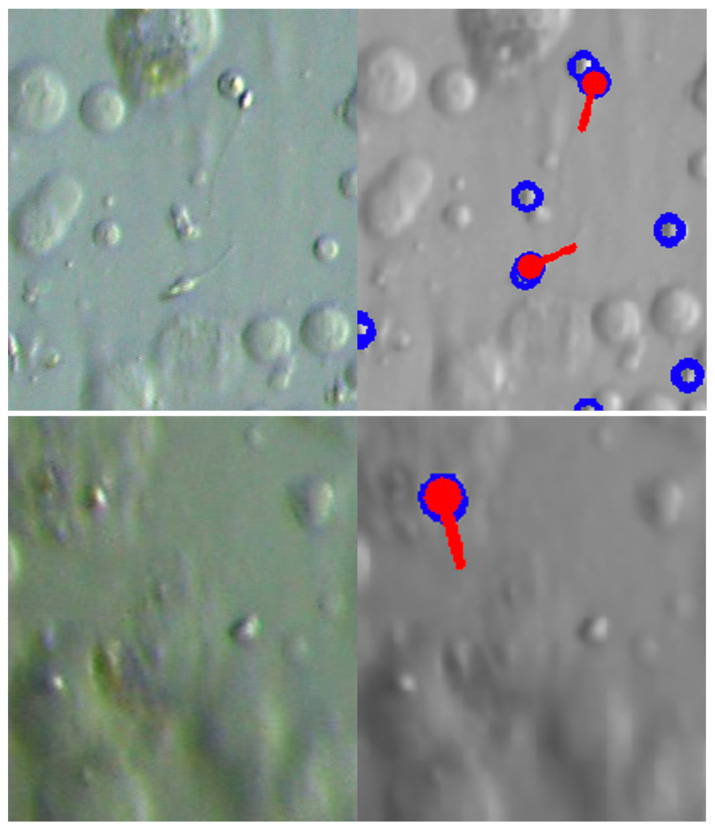
Detection example using SD-CLIP. The blue circles show the potential sperm heads and the red shapes show the detected sperm.

SD-CLIP [12] can estimate the orientation of the sperm head and neck, as illustrated in Figure 5. In the present study, we use SD-CLIP to obtain the coordinates of sperm heads and use these as reference points for quantifying motility. Specifically, we consider displaying VSL values within the proposed system, defined as the distance between the sperm head position at a given time and its position one second later. Because sperm detection in Micro-TESE tissue is inherently challenging, and reliable detection in every frame cannot be guaranteed, it is difficult to robustly compute more complex kinematic indices. For this reason, we focus on VSL as a feasible and informative index for motility evaluation in this context.

### 2.7. Evaluation Metrics

In previous sperm detection models such as those in [11,12], multiple sperm head coordinates are output, and correctness is evaluated manually through visual inspection. Based on whether each detected head position is correct, the detection performance can be evaluated using Precision, Recall, and their harmonic mean (F-value). In contrast, object detection models such as YOLOv5 express object locations using bounding boxes. The degree of overlap between a predicted bounding box $B_{pre}$ and the ground-truth bounding box $B_{gt}$ is evaluated using the Intersection over Union (IoU) metric:

(1) $$IoU=\frac{area\left(B_{pre}\;\cap \;B_{gt}\right)}{area\left(B_{pre}\;\cup \;B_{gt}\right)}$$

For example, even if two identical squares are shifted by one-ninth of their diagonal length, the IoU drops to approximately 0.65, indicating sensitivity to positional deviations. In this study, detections with IoU > 0.45 were considered correct. Precision, Recall, and F-value were calculated accordingly.

Object detection models also depend on a confidence threshold. Increasing the threshold reduces false positives but increases false negatives; decreasing it does the opposite. This trade-off is visualized using a Precision–Recall (PR) curve. The closer the curve is to the upper-right corner, the better the detection performance. The Average Precision (AP) is defined as the area under the PR curve:

(2) $$AP=\int_{0}^{1}p(r)dr$$

Since only discrete data are available, AP is approximated using:

(3) $$AP=\sum_{i=2}^{N}\left(r_{i}-r_{i-1})P(r_{i}\right)$$

The mean Average Precision (mAP) is defined as:

(4) $$mAP=\frac{1}{C}\sum_{i=1}^{C}AP_{i}$$

Since only one class (sperm) is detected in this study, AP and mAP are identical.

## 3. Results and Discussion

### 3.1. Evaluation of the AR-Display Microscope System

To assess the positional accuracy of the AR overlay, a mesh-like device featuring square apertures of 0.25 mm arranged in a grid with 0.5 mm spacing both vertically and horizontally was prepared [33]. A task was then performed in which the apertures of this microdevice were identified and displayed using the microscope. To capture the microscopic field images, the following settings were applied to the high-speed USB camera in order to shorten the exposure time:

• 2 × 2 binning in both vertical and horizontal directions
• Gain set to 128

Binning is a sensitivity enhancement function in which multiple pixel charges are combined and output as one pixel. With 2 × 2 binning, four pixels (2 × 2) are treated as a single pixel, increasing sensitivity by a factor of four while reducing spatial resolution to one quarter. As a result, the camera image resolution was reduced to 1224 × 1024 pixels. Gain refers to the amplification of the electrical signal converted from incoming light at the imaging sensor. It is used when exposure cannot be sufficiently increased through shutter speed or aperture adjustments due to light limitations.

**Figure 6:**
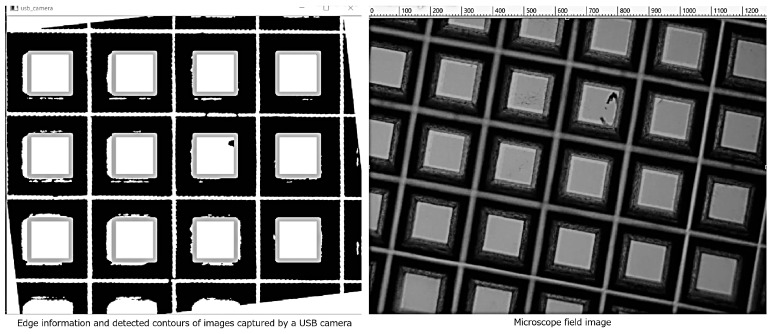
AR display of the bottom area of the microwell array. Spatially aligned display was confirmed.

Figure 6 shows the visualization of the bottom surface of the microdevice using images captured at 10 FPS from the high-speed USB camera. Although the camera supports 75.7 FPS, the actual operation speed was 10 FPS because an exposure time of approximately 100 ms was required to achieve sufficient brightness for human visual observation.

This confirms that the complete processing flow, from image acquisition by the high-speed camera, image processing, to information overlay on the microdisplay, operates correctly and produces valid AR visualization. The AR overlay was also confirmed to align accurately with the microscopic field of view. However, one limitation of the AR-display microscope system is that the brightness of the microdisplay is relatively low, requiring the microscopic field to be darkened to some extent for the AR information to remain visible. Although this brightness is within a tolerable range for human vision, it is slightly dim for optimal observation and necessitates an exposure time of approximately 100 ms for image acquisition. Despite applying various image enhancement settings, the brightness of the microdisplay currently remains the primary bottleneck limiting the achievable AR frame rate.

### 3.2. Evaluation of the Sperm Detection Model

#### 3.2.1. Training Conditions for the Sperm Detection Model

YOLOv5s was selected as a trade-off between detection accuracy and real-time inference speed, enabling deployment on a notebook-class GPU required for intraoperative AR visualization. Nineteen videos of Micro-TESE microscope images (1920 × 1080 pixels) were used as training data. Frames were sampled every five frames and manually annotated. For each video, 12 were assigned for training, 2 for validation, and 5 for testing. Training conditions for YOLOv5 were as follows:

• Model: YOLOv5s
• Epochs: 220
• Image size: 640
• Optimizer: Stochastic Gradient Descent (SGD)

The model with the lowest validation loss was selected for evaluation.

#### 3.2.2. Evaluation Results of the YOLOv5 Sperm Detection Model

**Figure 7:**
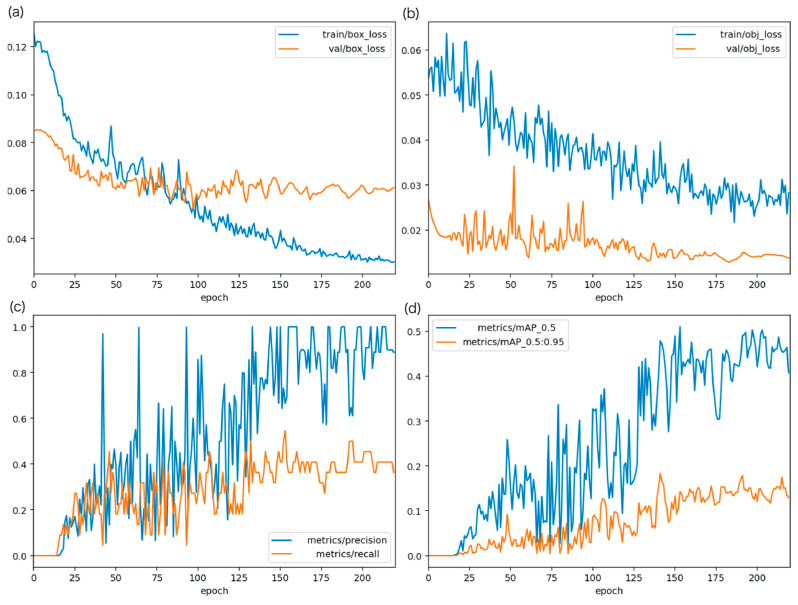
Training progress of the sperm detection model using YOLOv5: changes in (**a**) box loss, (**b**) object loss, (**c**) precision and recall, and (**d**) mAP with increasing epochs. All performance metrics improve steadily as training proceeds.

Figure 7 shows epoch-wise changes in (a) box loss, (b) object loss, (c) Precision/Recall, and (d) mAP. All indicators improved steadily as training progressed.

**Figure 8:**
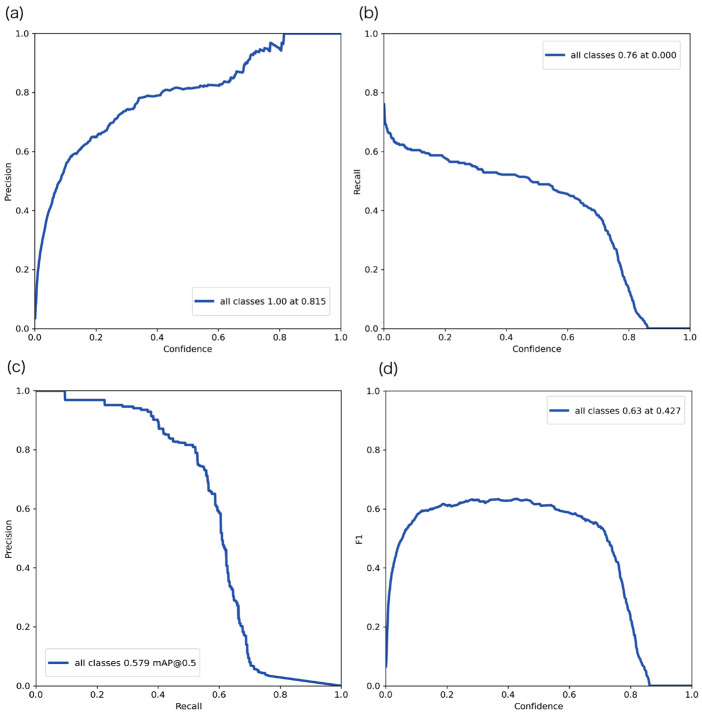
Evaluation results of the YOLOv5-based sperm detection model using the test dataset: (**a**) Precision curve, (**b**) Recall curve, (**c**) Precision–Recall (PR) curve, and (**d**) F-value curve.

Figure 8 shows the Precision, Recall, PR, and F-value curves on the test dataset. The F-value remained near 0.6 across different thresholds, indicating stable performance. The model was particularly stable when the confidence threshold ranged from 0.2 to 0.6.

#### 3.2.3. Comparison with Previous Detection Methods

Table 1 compares the proposed YOLOv5-based sperm detection model with previous methods [11,12]. YOLOv5 achieved the highest Precision and F-value. The runtime was 31 ms on a laptop GPU, well within the 100 ms target, confirming real-time applicability and satisfying performance requirements.

The performance gap for SD-CLIP is attributed to differences in datasets; [12] used mouse testicular samples, whereas this study used provisional Micro-TESE samples.

There is room for improvement in recall, but, at present, precision and recall have a trade-off relationship. This study aims to reduce the workload of operators in Micro-TESE procedures. If precision is low, operators would need to repeatedly verify the results, which would not alleviate their workload. Therefore, we tuned the system to achieve a precision of at least 0.8.

#### 3.2.4. Examples of Actual Detection Results of the Proposed Model

**Figure 9:**
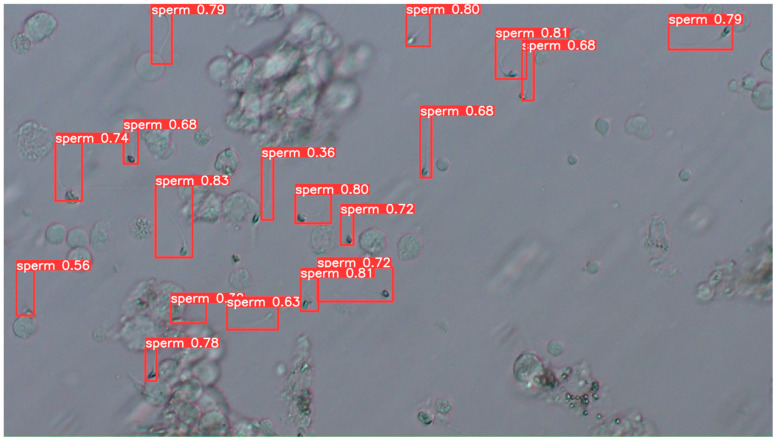
Example of detection results using test data 1. It can be seen that many sperm are detected cleanly as rectangles from head to tail with high confidence.

**Figure 10:**
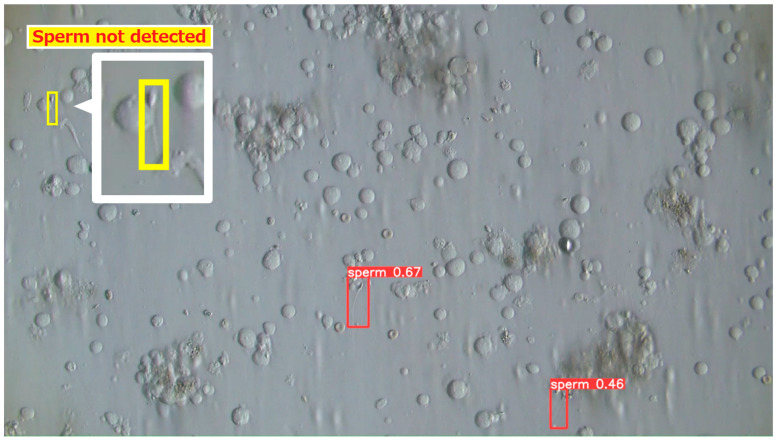
Example 2 of detection results using test data. Detection failed for the sperm enclosed in the yellow rectangle at the top left. Sperm tissue overlapped the tail region, likely preventing the model from recognizing its shape.

**Figure 11:**
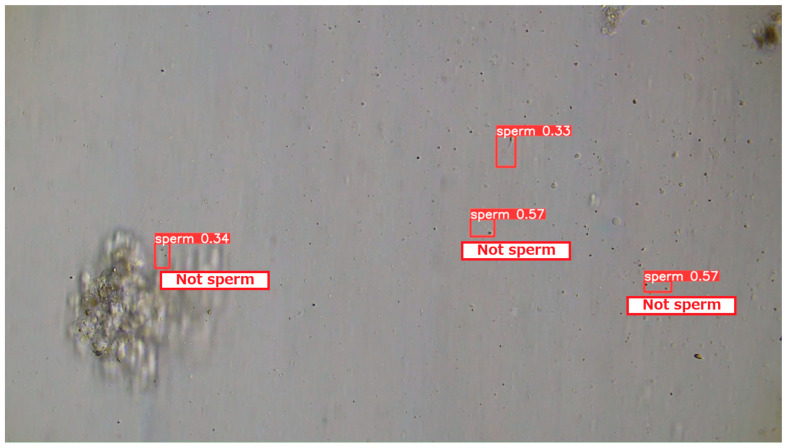
Example 3 of detection results using test data. Three cases exist where non-sperm objects were misdetected as sperm. Sperm-like tissue resembling a sperm head exists at the edge of the rectangle, suggesting the model attempts to detect sperm-like objects.

The detection results obtained on the test dataset are shown in Figure 9, Figure 10 and Figure 11. Figure 9 presents a representative example of successful sperm detection. Although a large number of sperm cells are present in the image, no false detections of surrounding tissue structures are observed, and all visible sperm are correctly detected. Figure 10 illustrates a typical case of missed detection. In this example, part of the sperm body is occluded by surrounding tissue, and the overall shape of the sperm cannot be clearly observed. Such occlusions are considered to be a major cause of detection failure. A possible solution to improve robustness is to augment the training dataset with a larger number of samples in which sperm are partially covered by tissue structures. Figure 11 shows an example of false-positive detection. In the lower-left region of the image, tissue structures arranged along a curved shape are incorrectly detected as sperm. In other false-positive regions, elliptical tissue fragments resembling sperm heads appear near the edges of the detected bounding boxes. These results indicate that the model tends to respond to sperm-like geometric patterns, even when the detected objects are not true sperm cells.

### 3.3. Evaluation of Sperm Dynamic Analysis

**Figure 12:**
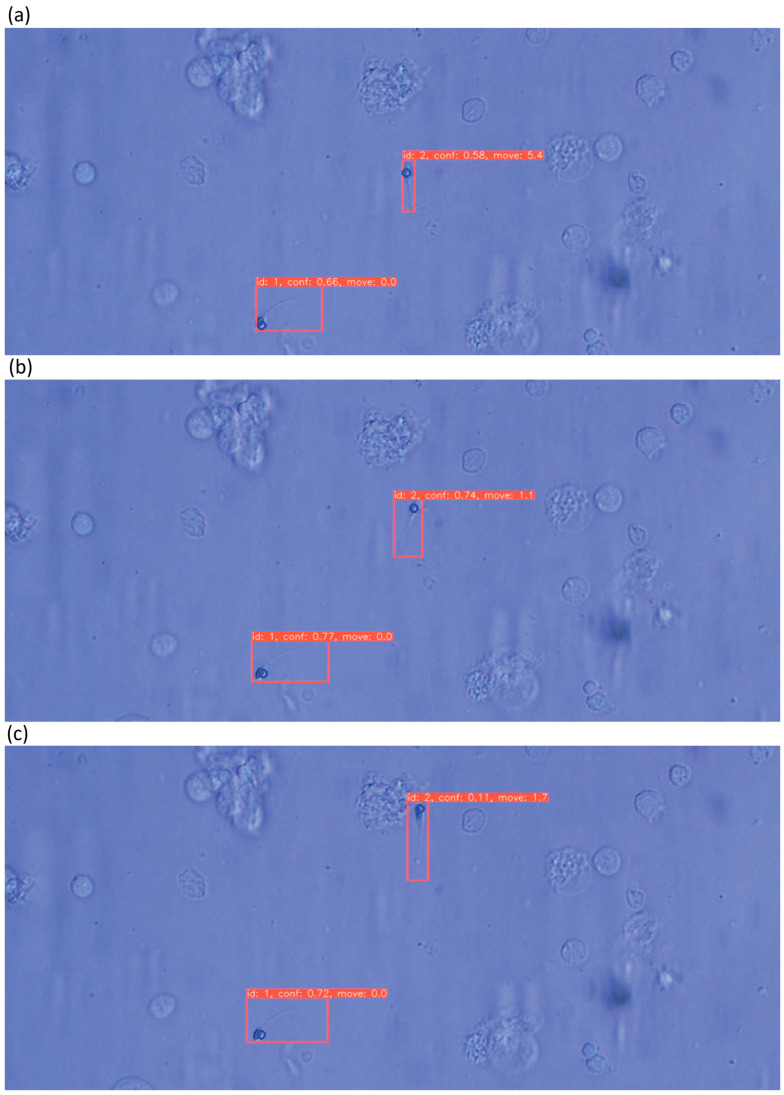
Image showing sperm tracking using DeepSORT with assigned IDs, head detection using SDCLIP (indicated by blue circles), calculated momentum, and each piece of information overlaid on the microscope field-of-view image. Both tracking and momentum display are successful. Subfigures (**a**–**c**) show the detection results at different time-frames.

**Figure 13:**
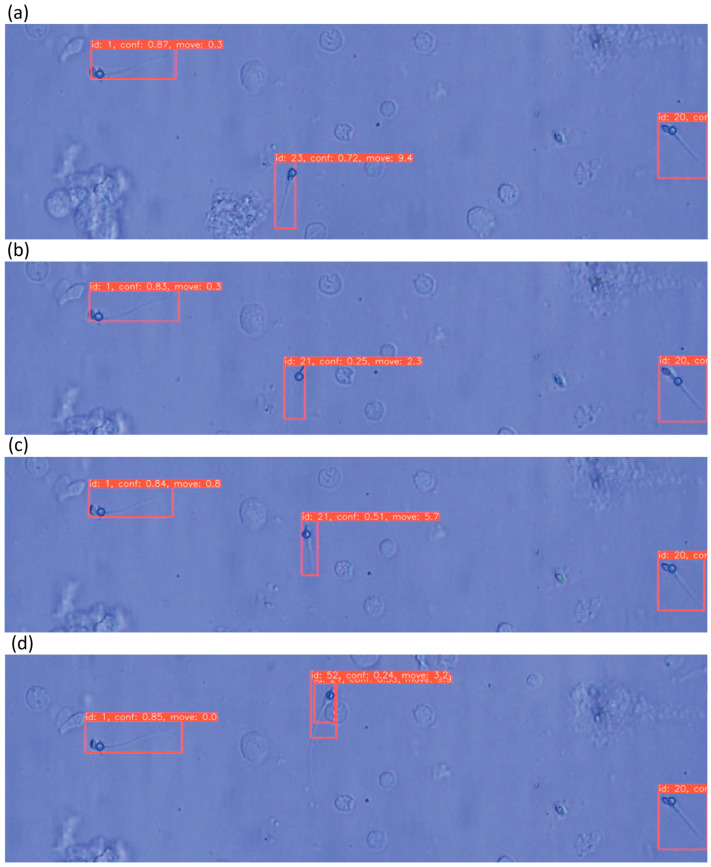
Image showing sperm tracked using DeepSORT with assigned IDs, heads detected using SD-CLIP (indicated by blue circles), and momentum calculated. Each piece of information is overlaid on the microscope field-of-view image. Tracking ID assignment failed in some cases, and double rectangles were predicted, indicating unsuccessful tracking. Subfigures (**a**–**d**) show the detection results at different time-frames, where double detection happened at (**d**), which is considered a failure.

Tracking results using DeepSORT are shown in Figure 12 and Figure 13. Tracking IDs were correctly assigned with a runtime of 31 ms. Velocity estimates reflected actual movement correctly. However, ID switches occurred when sperm rotated significantly or when YOLOv5 produced duplicate detections.

### 3.4. Evaluation of the Entire Proposed System

**Figure 14:**
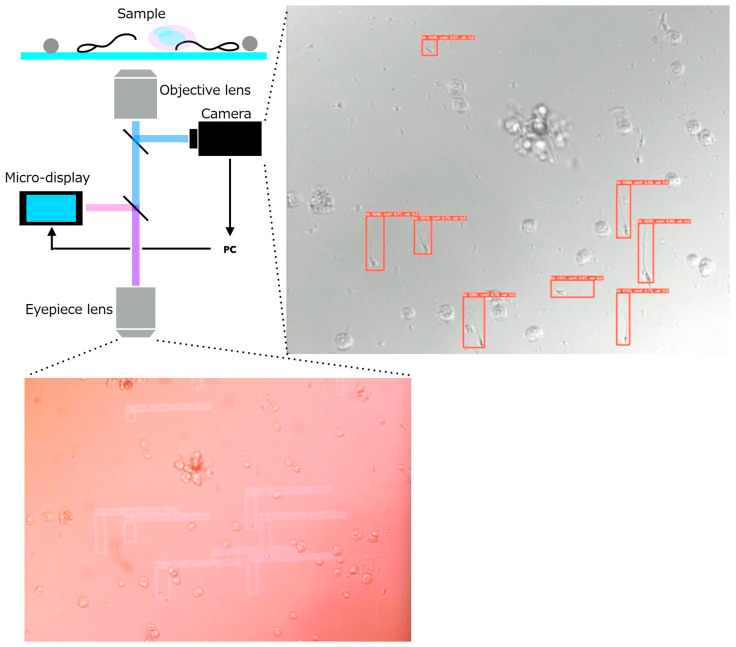
Microscopic field image with AR-overlaid sperm positions and the corresponding output image obtained by applying the sperm detection model to the USB camera input.

A test sample containing sperm extracted from testicular tissue was prepared, and the AR display of sperm position, tracking ID, and velocity information within the microscopic field of view was evaluated. Figure 14 shows the microscopic field of view with AR-based sperm visualization, together with the corresponding image obtained by applying the sperm detection model to the USB camera input. These results demonstrate that the integration of the AR microscope system and the sperm analysis software was successfully achieved. Specifically, the complete workflow from acquiring microscopic images, analyzing sperm information based on the captured images, and displaying the extracted information on the microdisplay was confirmed to operate correctly (See Supplementary Video S1).

**Figure 15:**
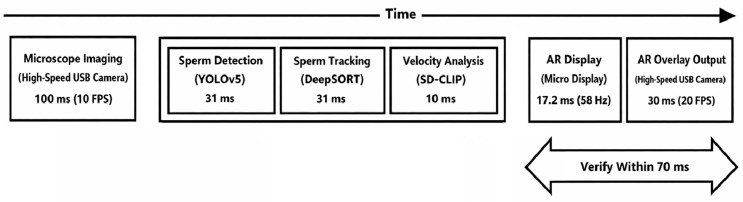
Schematic illustration of the processing latency in the proposed system. The diagram shows the time required for each component of the pipeline.

Next, the total system latency, defined as the time elapsed from microscopic image acquisition to the AR visualization of sperm information, is evaluated. A schematic breakdown of the processing time for each system component is shown in Figure 15. Based on the time difference between the output of the sperm analysis software and the AR-overlayed microscope images, the delay from sperm analysis completion to projection was confirmed to be within 70 ms.

Although precise measurement of the total system latency is technically challenging and remains a topic for future work, the overall system delay can be approximately estimated as follows:

(5) $$\begin{array}{c} Total\;delay\approx 100+\left(camera\;image\;transfer\;time\right)+31+31+10+\left(70-30\right)\\ \approx \left(camera\;image\;transfer\;time\right)+212\;\mathrm{ms} \end{array}$$

## 4. Conclusions and Future Work

In this study, we developed a system to accelerate the workflow of embryologists during Micro-TESE by supporting the identification and retrieval of spermatozoa under a microscope. Using an AR-assisted microscope, we constructed a support system that overlays real-time visual guidance during sperm search operations. The system consists of the following components:

• Construction of an AR microscope system
• Development of a real-time sperm analysis software suite, including:
  ○ Sperm detection
  ○ Sperm tracking
  ○ Sperm velocity estimation

Each module was implemented and evaluated.

For the AR microscope, we verified that the complete pipeline—from acquisition of microscopic images using a high-speed camera to AR overlay via the microdisplay—operated at 10 FPS. For the sperm analysis software, YOLOv5 achieved a Precision of 0.81, Recall of 0.52, F-value of 0.64, and an execution time of 31 ms. This F-value exceeded that of prior sperm-detection methods, and the runtime satisfied real-time requirements. Using DeepSORT, we successfully assigned tracking IDs to individual spermatozoa and achieved stable multi-object tracking. Furthermore, by applying the SD-CLIP head-candidate detection algorithm, we extracted head coordinates over time and computed VSL as an index of motility. Our system achieved basic motility measurement, but morphological evaluation was not yet implemented; this remains a topic for future work.

As future directions, improving the user experience of the AR microscope is important. One limitation during this study was the brightness of the microdisplay, which constrained the achievable FPS. Higher-brightness microdisplays could increase system frame rate and AR clarity. For the sperm analysis software, further improvement in detection, tracking, and head-coordinate estimation accuracy will be necessary. With these enhancements, additional motility metrics such as VCL and VAP, as well as morphological evaluation, could be incorporated—strengthening the system’s capability to recommend high-quality spermatozoa for clinical use.

In this paper, due to ethical constraints, HepG2 cells were used instead of actual clinical Micro-TESE samples. HepG2 cells tend to aggregate when collected from culture containers, resulting in a suspension containing objects of various sizes, from single cells to clusters of several hundred cells. This approach reproduces conditions similar to actual clinical Micro-TESE samples; however, it does not fully mimic the complexity of real samples, such as blood vessels and blood. Therefore, validation using authentic clinical Micro-TESE samples remains an important subject for future work.

Other aspects to consider in future work are continual or adaptive learning strategies. It may be incorporated to mitigate performance drift caused by variability in imaging conditions, microscope settings, and specimen characteristics over time. Updating the detection model using newly acquired clinical data could improve robustness and long-term applicability of the system, as demonstrated in recent adaptive learning studies [34,35]. Such approaches are particularly relevant for deployment in real clinical environments, where static models may degrade over extended use.

## Figures and Tables

**Table 1:** Performance comparison on 32 Micro-TESE microscope images (* indicates values from literature).

Method	Precision	Recall	F-Value	Runtime (ms)
AKAZE + MB-LBP [11] *	0.38	0.87	0.53	-
SD-CLIP [12]	0.28 (0.75)	0.26 (0.58)	0.27 (0.65)	47
YOLOv5	0.81	0.52	0.64	31

## Data Availability

The original contributions presented in this study are included in the article/Supplementary Materials. Further inquiries can be directed to the corresponding author.

## Supplementary Materials

The following supporting information can be downloaded at: https://www.mdpi.com/article/10.3390/bioengineering13010102/s1, Video S1: the real-time output of the proposed augmented-reality microscope system.

## Abbreviations

The following abbreviations are used in this manuscript:

ART	Assisted reproductive technology
ET	Embryo transfer
IVF	in vitro fertilization
ICSI	Intracytoplasmic sperm injection
FET	Frozen–thawed embryo transfers
Micro-TESE	Microdissection testicular sperm extraction
NOA	Non-obstructive azoospermia
AR	Augmented reality
ARM	Augmented reality microscope
FPS	Frames per second
SD-CLIP	Sperm Detection using Classical Image Processing
YOLO	You Only Look Once
SSD	Single Shot MultiBox Detector
CASA	Computer-Aided Sperm Analysis
VCL	Curvilinear velocity
VSL	Straight-line velocity
VAP	Average path velocity
ALH	Amplitude of lateral head displacement
LIN	Linearity
WOB	Wobble
STR	straightness
BCF	Beat-cross frequency
MAD	Mean angular displacement
MOT	Multi-Object Tracking
SORT	Simple Online and Realtime Tracking
IoU	Intersection over Union
PR	Precision–Recall
AP	Average Precision
mAP	mean Average Precision
SGD	Stochastic Gradient Descent
